# CRISPR/Cas9-mediated knockout of intracellular molecule SHP-1 enhances tumor-killing ability of CD133-targeted CAR T cells in vitro

**DOI:** 10.1186/s40164-023-00450-x

**Published:** 2023-10-06

**Authors:** Ming Liu, Linlin Zhang, Mingtian Zhong, Yihao Long, Wenhui Yang, Ting Liu, Xingxu Huang, Xiaodong Ma

**Affiliations:** 1 National Center for Respiratory Medicine, National Clinical Research Center for Respiratory Disease, State Key Laboratory of Respiratory Disease, Guangzhou Institute of Respiratory Health, The First Affiliated Hospital of Guangzhou Medical University, Guangzhou, 510120 China; 2 Model Animal Research Center, MOE Key Laboratory of Model Animal for Disease Study, Nanjing University, Nanjing, China; 3 Key Laboratory of Brain, Cognition and Education Sciences, Institute for Brain Research and Rehabilitation, Guangdong Key Laboratory of Mental Health and Cognitive Science, Ministry of Education, South China Normal University, 510631 Guangzhou, China; 4 Zhejiang Lab, 311121 Hangzhou, Zhejiang China; 5 School of Life Science and Technology, ShanghaiTech University, 201210 Shanghai, China

**Keywords:** SHP-1, CD133, CAR, CRISPR/Cas9, PiggyBac Transposase

## Abstract

CAR T cell therapy has been successfully used in the treatment of hematological malignancies, and the strategy that deletion of inhibitory receptor on the CAR T cell surface, such as PD-1, greatly enhance the antitumor effects. Here, we describe a one-step electroporation for the co-transfection of Cas9:sgRNA and CAR plasmids on primary T cells to demonstrate the effect of SHP-1 deletion in CAR T cells. By using PiggyBac Transposase system, we can achieve more than 90% of T cells express CAR gene and nearly 60% SHP-1 knockout efficiency in T cells. We show that knockout of SHP-1 in CD133 CAR T cells resulted in significantly improve the cytolysis effect on CD133 positive glioma cell lines. We further demonstrate that the enhanced antitumor efficacy of SHP-1 deletion is due to the increased release of TNF-α, IL-2 and IFN-γ in vitro. Finally, we evaluated the biosafety of Cas9 genome editing and did not find any insertions of Cas9 and obvious editing in off-target sites in CAR T cells. These data provide an approach for achieving both intracellular inhibitory molecule, SHP-1 deletion and CD133 CAR gene over-expression in human T cells. And SHP-1 could be a new potential target for adoptive CAR T cells immunotherapy.

**Supplementary Information:**

The online version contains supplementary material available at 10.1186/s40164-023-00450-x.

## To the editor

**Fig. 1:**
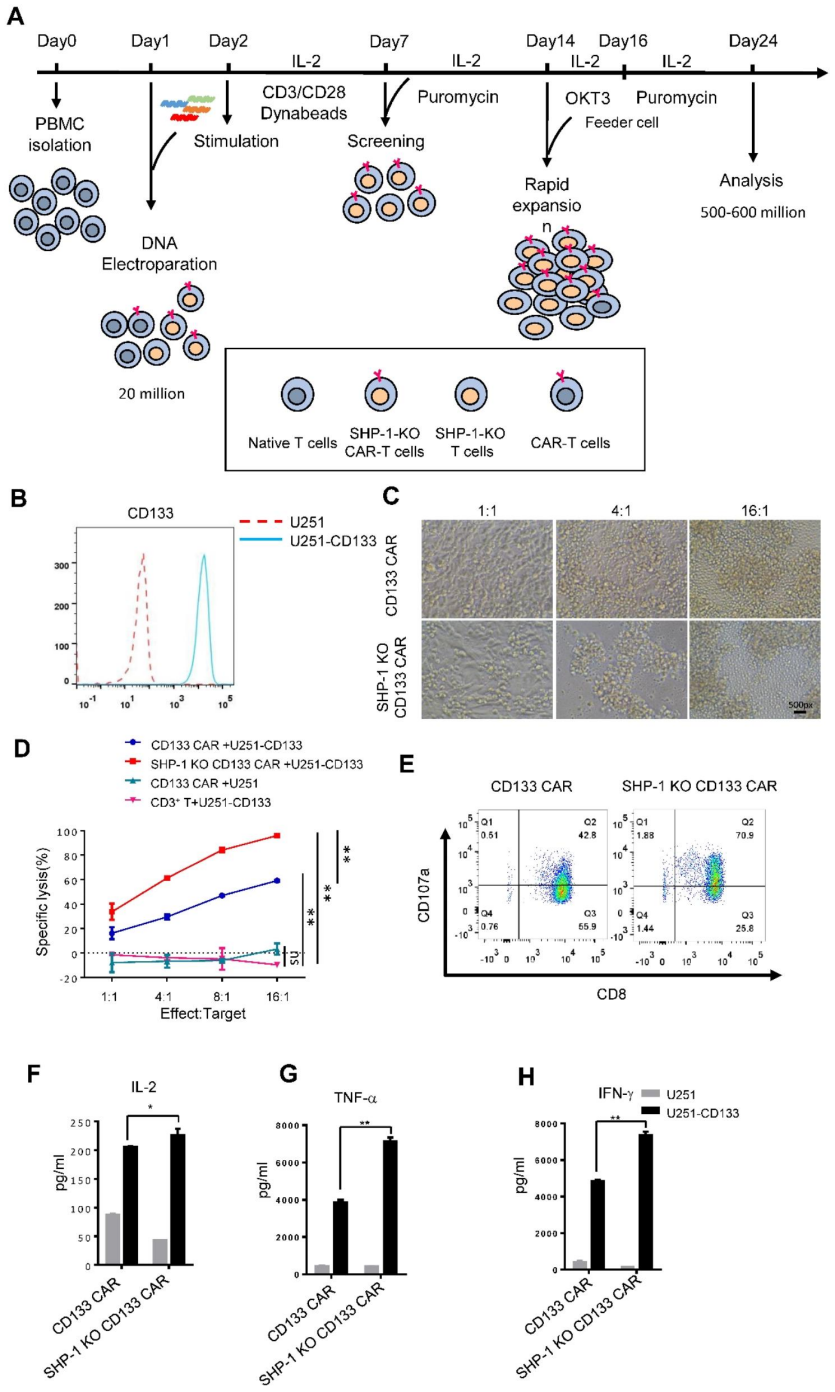
The disruption of SHP-1 enhanced the cytolysis of CAR T cells in vitro. (A)Optimize the protocol of gene knockout CAR-T cells. **(B)** U251 cells were transfected with CD133 cDNA, and CD133 expression on cell surface was evaluated by flow cytometry. **(C)** The CD133 CAR T and SHP-1 KO CD133 CAR T cells were co-cultured with U251-CD133 target cells at the effect-to-target ratios of 1:1, 4:1 and 16:1 respectively, and the survival of target cells were observed after 16 h. **(D)** The indicated CAR-T cells and target tumor cells were cocultured for 16 h at the effector-to-target ratios of 1:1, 4:1, 8:1 and 16:1 respectively, and the lysis of target cells were measured. **(E)** The expression of CD107a on the cell membrane of CD133 CAR-T and SHP-1 KO CD133 CAR T cells under the stimulation of CD3 antibody were evaluated by flow cytometry. **(F)-(H)** The supernatant was collected after CD133 CAR-T cells or SHP-1 KO CD133 CAR T cells were co-cultured with CD133 negative U251 cells (U251) or CD133 positive U251 cells (U251-CD133) for 16 h, and the levels of IL-2 (E), TNFα (F) and INF-γ (G) cytokines were determined by ELISA assay. *P < 0.05 or **P < 0.01 indicates a significant difference between the indicated groups (n = 3, two-way ANOVA in D and one-way ANOVA in F, G and H, and Tukey posttest). ns, not significant

SHP-1, encoded by the *PTPN6* gene, is a widely expressed inhibitory protein tyrosine phosphatase and is the most studied phosphatase in T cells [1]. Numerous studies have shown that SHP-1 plays a negative regulatory role during the activation and proliferation of antigen-dependent T cells [2]. Specific knockout of SHP-1 in T cells has been found in both blood and solid tumor mouse models to increase activation of T cells [3, 4], which confirmed that knockout of SHP-1 significantly improves the ability of T cells to kill tumor cells [5, 6]. Here, we used one step electroporation for the co-transfection of Cas9:sgRNA and CAR expression plasmids on primary T cells to demonstrate the effect of SHP-1 deletion in CAR T cells (Fig. 1A). First, we confirmed our optimized protocol for efficient knockout SHP-1 and expression of CAR on T cells from all three donors in one reaction (Supplementary Fig. 1A-1G) [7]. Next, to investigate the effect of SHP-1 knockout on CD133 CAR T cells function, we constructed human glioblastoma cell line (U251) that overexpressed CD133 antigen as a target cell (Fig. 1B). We co-cultured the WT and SHP-1 knockout CAR T cells with the target cells, and the killing effect of SHP-1 knockout CAR T cell was increased by nearly 40% compared with WT CAR T cells (Fig. 1C and D). Furthermore, we also measured the killing effect of CAR T cells on non-target cells, U251 without CD133 transfection (U251), and the results shown that CD133 CAR T had no killing effect on the U251 cells (Fig. 1D). After tumor cell co-culture, CD107a expression was increased on CAR T cells with SHP-1 knockout compared to WT controls (Fig. 1E). Moreover, a marked increase in IL-2, TNF-α, and IFN-γ was observed in SHP-1-deletion CD133 CAR T cells (Fig. 1F H). We also found that the proportion, activation and differentiation of CD133 CAR T cells with SHP-1 deletion were not altered (Supplementary Fig. 2A-2 H). These data indicate that enhanced cytotoxicity of CD133 CAR T cells with SHP-1 knockout is partially due to the elevation of cytokine releasing.

**Fig. 2:**
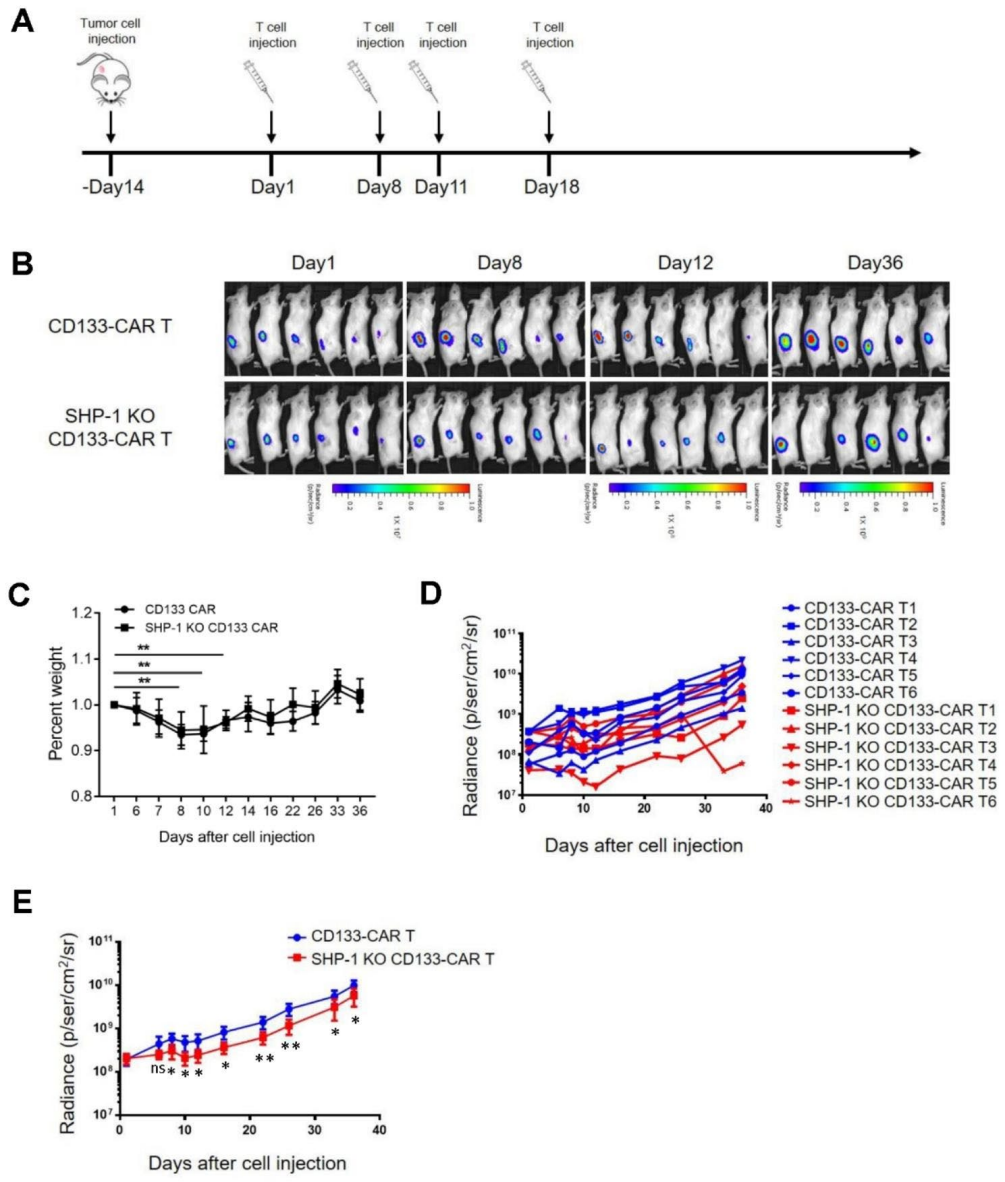
The disruption of SHP-1 enhanced the cytolysis of CAR T cells *in vivo.* **(A)** Schematic diagram of CAR-T cell therapeutic mouse model construction. 1 × 10^6^ target cells (U251-CD133-luc) were seeded subcutaneously on the back of NPG mice. 2 × 10^6^ CAR T cells infusion therapy was performed on Day1, Day8, Day11, and Day18, respectively, and imaging tracking of tumor sizes were performed every 2–3 days. **(B)** Tumor size were detected by using in vivo imaging systems during CAR T cell treatment. The time point of the first T cell therapy was recorded as Day1, and the tumor growth in mice was recorded on Day1, Day8, Day11, and Day18, respectively. **(C)** Body weight were measured in mice treated with CD133 CAR T cell and SHP-1 KO CD133 CAR T cells. **(D)** Tumor size were evaluated by quantified the areas of luciferase signal. Each curve represents the luminescence signal value of tumor in one mouse. **(E)** The mean value of luminescence signal of tumors in mice treated with CD133 CAR T cells and SHP-1 KO CD133 CAR T cells were evaluated. **P < 0.01 indicates a significant difference between the different time points (n = 6, one-way ANOVA in C). *P < 0.05 or **P < 0.01 indicates a significant difference between CD133 CAR T cells and SHP-1 KO CD133 CAR T cells (n = 6, one-way ANOVA in E, and Tukey posttest). ns, not significant

Next, we measured the tumor killing ability of CD133 CAR T cells with or without SHP-1 deletion using immunodeficiency NPG mice with overexpressing CD133 and luciferase U251 tumor cell (U251-CD133-luc) transplantation. The first day of CAR T cell injection was defined as Day1, and CAR T cells were injected at Day1, Day8, Day11 and Day18 respectively (Fig. 2A). As shown in Fig. 2B, tumors grew more slowly in mice receiving SHP-1 knockout CAR T cell therapy than in the non-knockout group. We further found that all mice showed significantly less body weight loss at day 10 after the CAR T cell transferred. Correspondingly, at Day 20 post CAR T cell transferred, the body weight started to recovery and increase. There were no significantly difference of body changes in mice between CD133 CAR T and CD133 CAR T with deletion of SHP-1 treatment (Fig. 2C and D). Similarly, the luciferin values in tumor cells in mice also showed the same results, with tumors growing more slowly in mice treated with SHP-1 knockout CAR T cells than in the non-knockout group (Fig. 2E). In the end, we preliminarily test the safety of SHP-1 gene editing by examining the cleavage of CRISPR/Cas9 at potential off-target sites. At one day of T cell electroporation, about 36% of CD133 CAR T cells were GFP-positive, and no GFP-positive cells were detected after 24 days of culture (Supplementary Fig. 3A). In addition, we selected the highest-scoring off-target site on the website (www.crispr.mit.edu) based on the sgRNA sequence targeting SHP-1 (Supplementary Fig. 3B). PCR product analysis showed that the electrophoresis band was single, and there was no difference between the SHP-1 knockout group and the control group, which indicate that no large fragments were lost at this site. For T7EN1 digestion analysis, all band was single and no small fragments of bands were found, which suggest that there was no off-target effect at the corresponding site (Supplementary Fig. 3C). For more accurately evaluating the off-target effect of CRISPR editing, GUIDE-Seq will use to test the safety of SHP-1 editing in future work [8]. Compared with virus-transduced CAR T cells, very few clinical trials have applied CAR T cells prepared using PiggyBac system, and more attention needs to be paid on the safety of novel vector systems in future clinical trials [9].

In conclusion, we describe a one-step electroporation for CRISPR/Cas9 editing in CAR T cells and further demonstrate the role of the intracellular molecule SHP-1 in CAR T cell therapy against glioma.

### Electronic supplementary material

Below is the link to the electronic supplementary material.

Supplementary Material 1

## Data Availability

The datasets used and/or analyzed during the current study are available from the corresponding author on reasonable request.

## Abbreviations

SHP-1: Src homology 2 domain-containing protein tyrosine phosphatase 1
PTPN6: protein tyrosine phosphatase non-receptor type 6
CRISPR: clustered regularly interspaced short palindromic repeats
Cas9: CRISPR-associated protein 9
CAR: chimeric antigen receptor
PBMC: peripheral blood mononuclear cells
scFv: single-chain antibody variable fragment
