# Supplementary material for: CRISPR/Cas9-mediated knockout of intracellular molecule SHP-1 enhances tumor-killing ability of CD133-targeted CAR T cells in vitro
Source: Exp Hematol Oncol. 2023 Oct 6;12:88. doi: 10.1186/s40164-023-00450-x (PMC10559533; doi:10.1186/s40164-023-00450-x)
Supplement: Supplementary file 1 — Supplementary Material 1 [file 40164_2023_450_MOESM1_ESM.docx]

**Supplementary data**

**Materials and methods**

**Construction of the vectors**

The pGL3-U6sgRNA-puro plasmid with U6 promoter (addgene #51133) was used to construct vectors that express sgRNA in vivo. On human SHP-1 genome exon 2, the target sequence is screened according to the principle of 5'-GN(19)NGG-3', and the sgRNA primers was synthesized. The oligonucleotide sequences used to construct sgRNA expression vectors were listed in Table S 1. The scFv (single-chain antibody variable fragment) sequence of CD133 CAR was derived from the single-stranded variable region of the AC133.1 monoclonal antibody [1]. Then, the synthesized sequence is reassembled into a PiggyBac vector with a third-generation CAR structure. In addition, to facilitate enrichment of transfected CAR-T cells, we connected the resistance gene of puromycin via T2A at the C-end of the above sequence, which eventually formed the PiggyBac-CD133 CAR-puro plasmid. The super transposase plasmid is gifted from Dr. Xuekai Zhu [2, 3].

**Cell culture and transfection**

1x10^8^ U251 cells were suspended with 5 μg PiggyBac-CD133 cDNA-puro, 5 μg PiggyBac-luciferase, and 5 μg super-transposases plasmids, and then transfected by Nucleofector 2B using the Amaxa Human Cell Line Kit V (Lonza-Amaxa, VCA-1003). After 24 h of electroporation, cells were positively selected with a final concentration of 2 μg/ml puromycin and 200 μg/ml G418 to obtain a U251 cell line (U251-CD133-luc) with stable expression of CD133 antigen and Luciferase.

**PBMC isolation, T cell culture**

Human peripheral blood mononuclear cells (PBMC) were isolated from fresh whole blood. Whole blood was provided by The First Affiliated Hospital of Guangzhou Medical University, and all blood sources come from healthy donors. PBMC was isolated using lymphocyte isolate Ficoll Paque PLUS (GE, 17144002) and resuspended in AIM-V medium (Gibco, 12055091).

**T cell electroporation, activation and expansion**

2 x 10^7^ freshly separated PBMC were resuspended with electroporation buffer containing 5 μg Cas9-EGFP, 5 μg pGL3-U6-hSHP-1sgRNA-puro, 5 μg PiggyBac-CD133 CAR-puro, 5 μg super-transposases, and the mixture were electroporated by using Lonza Nucleofector 2B with U-014 procedure and Amaxa Human T cells Nucleofector Kit. 24h after electroporation, electro-transferred cells were stimulated by anti-human CD3/CD28 Dynabeads (Invitrogen, 11132D) and amplified in medium with 300 U/ml IL-2 (Peprotech, 200-02-1000). After 5 days of stimulation, beads were removed and positive CAR T cells were screened with 0.5 μg/ml puromycin.

Rapid expansion of CAR T cells were performed at day 14 of CAR T cells culture. PBMC irradiated with 40 Gy X-rays are used as feeder cells during the CAR T cell expansion. Irradiated PBMC were coculture with CAR T cells at ratio of 20:1, and 50 ng/ml anti-CD3 (OKT3, eBioscience, 4305130) and 300 U/ml IL-2 were added. After 48h co-incubation, anti-CD3 antibody was removed and cells were continually cultured with 300 U/ml IL-2 and 0.5 μg/ml puromycin for enriching positive CAR T cells.

**T7EN1 cleavage and sequencing**

1 x 10^6^ T cells were resuspended in lysis buffer (containing 10 μM Tris-HCl, 0.4 μM NaCl, 2 μM EDTA, and 1% SDS) with 20 μg/ml of proteinase K (Yeasen, 10401ES60) and digest overnight in a 55 °C water bath. Genomic DNA was extracted follow the protocol of phenol chloroform and ethanol precipitation. PCR were performed with Phanta® Max Super-Fidelity DNA Polymerase (Vazyme, P505-d3) to amplify DNA fragments containing sgRNA-targeted sequences, and the PCR product was then purified with AxyPrep PCR Clean up Kit (Axygen, AP-PCR-250). 200 ng purified PCR product was denatured and re-annealed in a thermal cycler. The hybrid products were digested with T7 endonuclease 1 (Vazyme, EN303-01/02) at 37 °C for 25min. PCR primers for amplifying the SHP-1-targeted region were listed in Table S2. The PCR products were ligated with pMD19T vector and sequenced by using M13F primer.

**Transcription**

Total RNA was extracted using Trizol (Invitrogen) from CAR-T cells and the qualities were determined by Agilent Bioanalyzer 2100. RNA was reverse transcribed with iScript Reverse Transcription Supermix kit (BIO-RAD). qPCR was performed using the SYBR Green probes and CFX96 Real-time PCR system (BIO-RAD).

**Flow cytometry**

Cells were stained with the following antibody: Myc-Tag mouse mAb (9B11, Cell Signaling), APC/CY7 Goat anti-mouse IgG (405316, Biolegend), Brilliant Violet 421™ Goat anti-mouse IgG (405213, Biolegend), PE anti-human CD133 (130-098-826, Miltenyi), PE/CY7 anti-human CD3 (344816, Biolegend), PE/CY5 anti-human CD8a (300910, Biolegend), PE anti-human CD4 (555347, BD), APC anti-human CD62L (559772, BD), PE anti-human CD45RO (555493, BD), APC anti-huamn CD28 (302912, Biolegend), PerCP/CY5.5 anti-human CD27 (356408, Biolegend), APC/CY7 anti-human CD25 (557753, BD) FITC anti-human CD107a (555800, BD). All events were acquired using CytoFLEX (Beckman Coulter) equipment according to standard procedures.

**Cell cytotoxicity assay**

The cytotoxicity of CAR-T cell was determined by using a luciferase-based assay as previously described. 2 x 10^5^ tumor cells were co-incubated with untranslated T cells, CAR T cells (CD133 CAR), and gene-edited CAR T cells (SHP-1 KO CD133 CAR) for 16h at effector-to-target ratios of 1:1, 4:1, 8:1 and 16:1. The cell morphology was observed under microscopy. And the Firefly Luciferase Reporter Gene Assay Kit (Beyotime) was used to measure luciferase activity of the remain tumor cells, and lysis was calculated as follows: 1-(N_1_-N_0_)/(N-N_0_) x 100%. N_0_, N, and N_1_ represent PBS, tumor cells only and tumor cells plus CAR-T cells, respectively.

**ELISA for cytokine expression**

To determine cytokine production, CAR-T cells were co-incubated with tumor cells at ratio of 16:1 for 16h. Cytokine concentrations in culture supernatant were measured using the IL-2, IFN-γ and TNFα ELISA kit (PerkinElmer) according to the manufacturer’s protocol.

**Xenograft mouse model**

NOD/Prkdc^scid^/IL-2Rγ^null^ (NPG) mice were obtained from Beijing Vitalstar Biotechnology (NOD-Prkdc^scid^ Il2rgtm1/Vst mice, stock No. VS-AM-001). 1 x 10^6^ U251-CD133-luc cells were subcutaneously injected to NPG mouse. Two weeks after tumor formation, mice were randomly divided into three groups: T cell group, CD133 CAR T cell group, and SHP-1 KO CD133 CAR T cell group. The initial day of CAR T cell transfer in situ was defined as Day1, and T cells were injected at Day1, Day8, Day11, and Day18 respectively, for a total of 4 treatments. The body weight of mice was measured every 2~3 days and tumor progression were detected using IVIS Spectrum Imaging System (Perkin Elmer).

**Statistical analyses**

Data were expressed as the mean ± standard deviation of at least three independent experiments. The results were statistically analyzed using a one-way ANOVA or two-way ANOVA analysis of variance for multiple-group comparisons and Tukey was used as post-hoc analysis. The statistical significance was set at a P value <0.05 or a P value <0.01.

**Supplementary Figures**


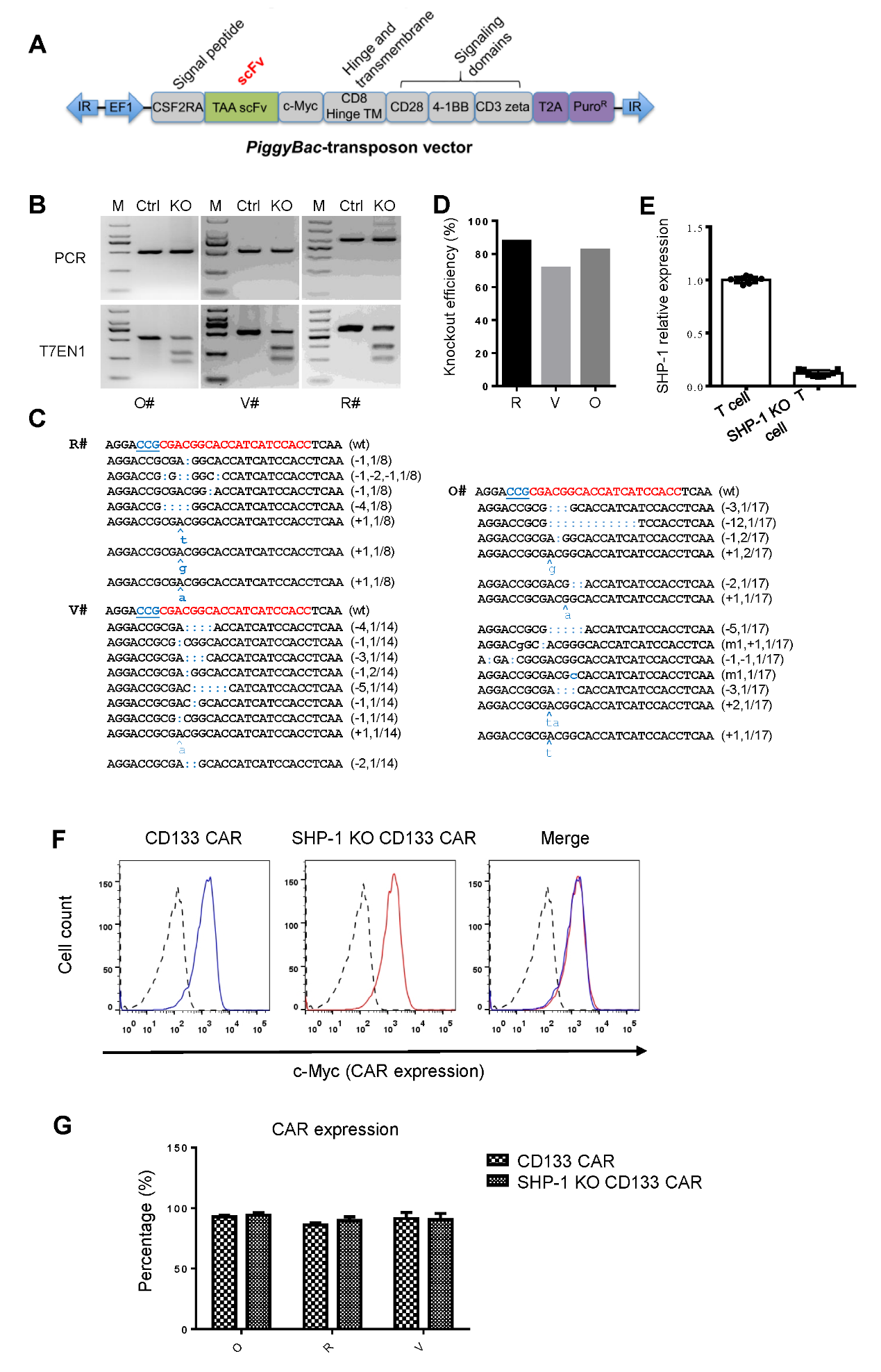


**Figure S1.** Highly efficient disruption of SHP-1 and CAR expression in CAR-T cells. **(A)** Schematic representation of CAR construct. CSF2RA is the signal peptide from colony-stimulating factor-2 receptor alpha. **(B)** Detection of sgRNA:Cas9-mediated cleavage of hSHP-1 by T7EN1 cleavage assay. Primary T cells were isolated from PBMC from three healthy donors and were electroporated with Cas9-EGFP, Hshp-1 sgRNA, PiggyBac-CD133 and super transposase. #O, #R and #V were defined as donor 1, donor 2 and donor 3 respectively. #O, #R (DL2000 DNA Marker) **,** #V (DL1000 DNA Marker)**. (C)** DNA sequences of SHP-1 knockout samples. TA clones from the PCR products were analyzed by DNA sequencing. The PAM sequences are underlined and highlighted in blue, the targeting sequences in red; the mutations in blue, lower case; deletions (−) and insertions (+). The above experiments have been repeated 3 times with similar results. SnapGene (4.1.8) was used to calculate indels frequence. **(D)** Knockout efficiency of SHP-1 in CAR-T cells from three healthy donors were evaluated by Sanger sequences. **(E)** SHP-1 expression in CAR-T cells with CTL-sgRNA-Cas9 and SHP-1-sgRNA-Cas9 were determined by qPCR. **(F)** Representative flow cytometry plot demonstrated CD133 CAR expression following CD3+ T cells electroporation. A c-myc tag is used for detection of scFv of CD133. **(G)** Percentage of positive CD133 CAR on CD3+ T cells from three healthy donors were evaluated.


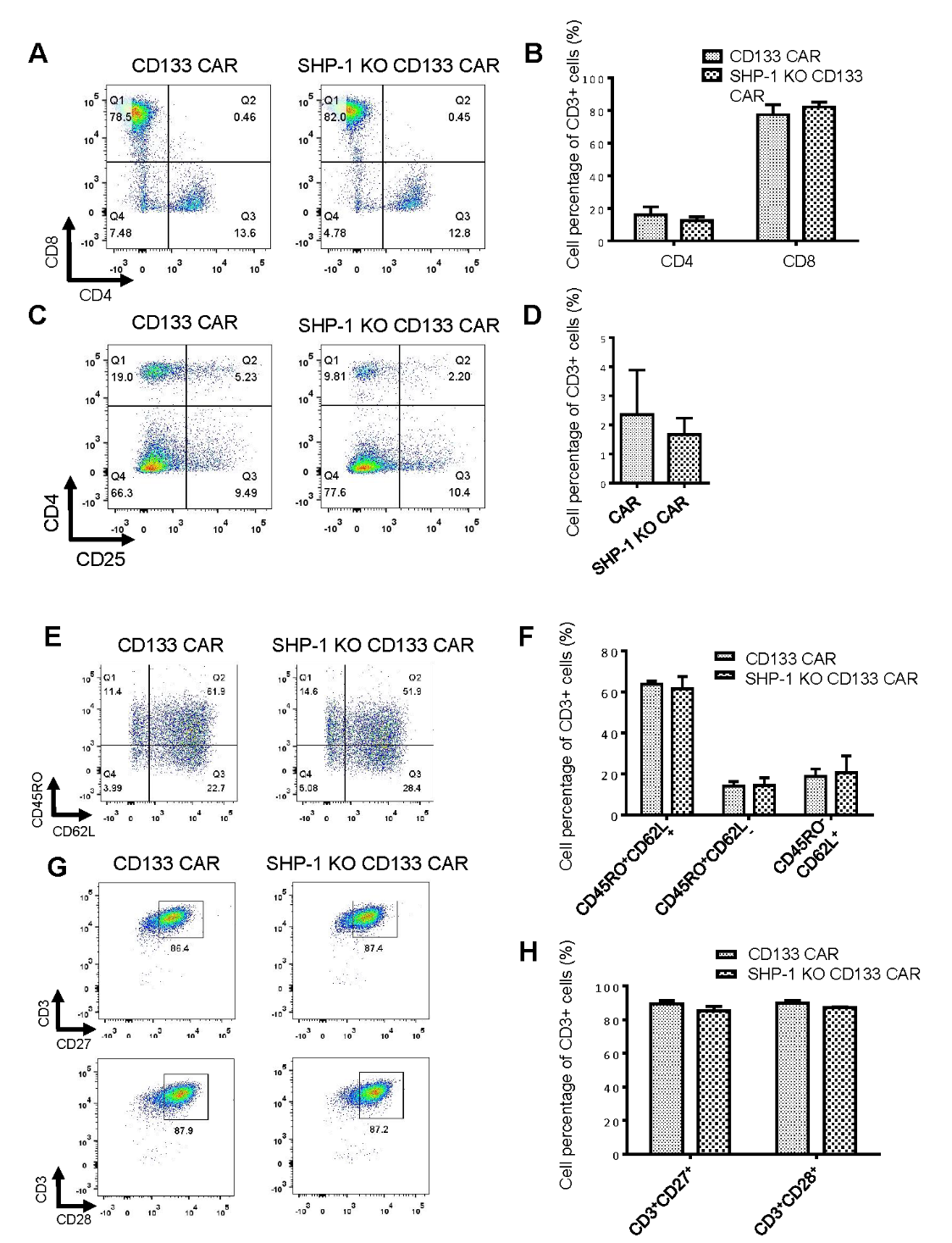


**Figure S2. SHP-1 knockout did not affect the phenotype of CAR T cells. (A)** Representative flow cytometry plot demonstrated the percentage of CD4^+^ and CD8^+^ T cells in CD133 CAR-T cells. **(B)** The percentage of CD3^+^;CD4^+^ and CD3^+^;CD8^+^ CD133-CAR-T cells were quantified from (A). **(C)** Representative flow cytometry plot demonstrated the percentage of CD4^+^ and CD25^+^ T cells in CD133 CAR-T cells. **(D)** The percentage of CD4^+^ and CD25^+^ CD133-CAR-T cells were quantified from (C). **(E)** Representative flow cytometry plot demonstrated the percentage of CD45RO^+^ and CD62L^+^ T cells in CD133 CAR-T cells. central memory T cells (CD45RO^+^;CD62L^+^); effector T cells (CD45RO^+^;CD62L^-^); and naïve T cells (CD45RO^-^;CD62L^+^). **(F)** The percentage of CD133-CAR-T cells stained with CD45RO and CD62L were quantified from (E). **(G)** Representative flow cytometry plot demonstrated the percentage of CD3^+^;CD27^+^ and CD3^+^;CD28^+^ T cells in CD133 CAR-T cells. **(H)** The percentage of CD133-CAR-T cells stained with CD27 and CD28 were quantified from (G).


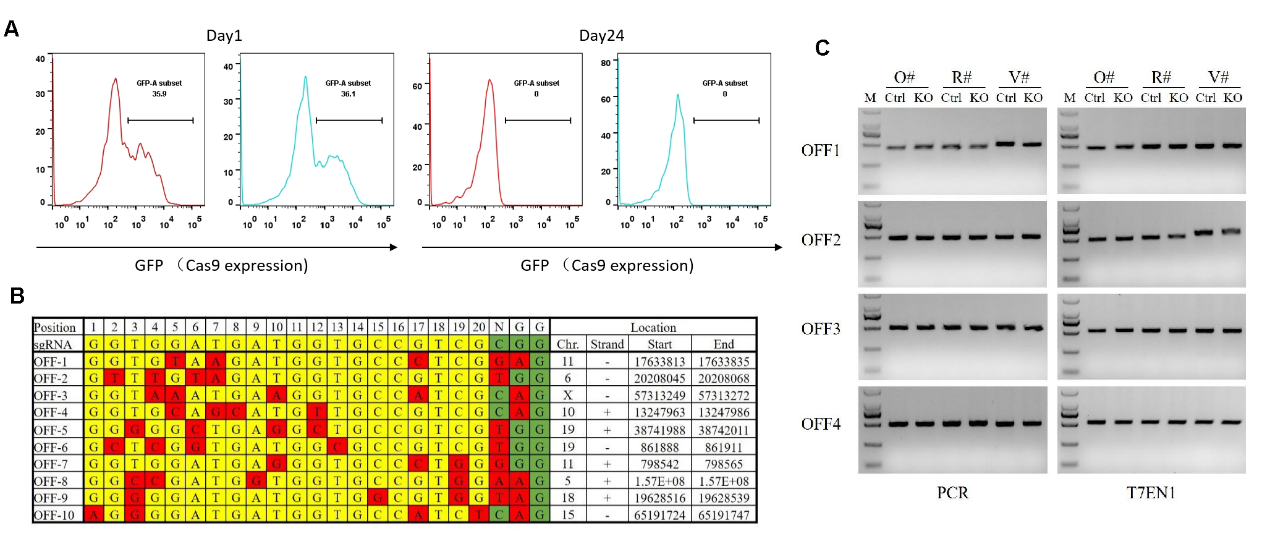
 **Figure S3. Off-target effects of CRISPR/Cas9 on CD133 CAR T cells. (A)** Cas9 expression in CAR T cells. Cas9 expression were evaluated by GFP intensities with flow cytometry. The red line represents the CD1333 CAR T cell, and the blue line represents the CD1333 CAR T cell with SHP-1 knockout. **(B)** The potential off-target site sequences of SHP-1. The yellow highlighted background indicates the same base as the sgRNA sequence, the green background represents PAM, the red background represents a base that is different from the sgRNA sequence, and the 20-base target sequence is numbered sequentially based on PAM distance. The position of the off-target sequence in the human genome is labeled on the right side. **(C)** The highest score of the first four off-target sites were evaluated by PCR and T7EN1 digestion, and the results showed no bands were cut.

**Supplementary Tables**

**Table S1. Oligonucleotide sequence of the sgRNA targeting SHP-1**

|  | |
| --- | --- |
| **Oligonucleotide name** | **Sequences** |
| hSHP-1-U6sg1 up | 5'-ACCGGTGGATGATGGTGCCGTCG-3' |
| hSHP-1-U6sg1 down | 3’-CACCTACTACCACGGCAGCCAAA-5' |
| hSHP-1-U6sg2 up | 5'-ACCGAGTTCTGGATCCGAATAT-3' |
| hSHP-1-U6sg2 down | 3'-TCAAGACCTAGGCTTATACAAA-5' |
| hSHP-1-U6sg3 up | 5'-ACCGGTGCCGTCGCGGTCCTGC-3' |
| hSHP-1-U6sg3 down | 3'-CACGGCAGCGCCAGGACGCAAA-5' |
| hSHP-1-U6sg4 up | 5'-ACCGTCACCCTGGTTCTTGCGACT-3' |
| hSHP-1-U6sg4 down | 3'-AGTGGGACCAAGAACGCTGACAAA-5' |

**Table S2. The primers for amplifying SHP-1sgRNA-targeted regions**

| **Primer name** | **Sequences** | **PCR products** |
| --- | --- | --- |
| hSHP-1-T7EN1 For1 | 5'-ACTCCCCGTCTGTTCCCTT-3' | 504bp |
| hSHP-1-T7EN1 Rev1 | 5'-GGGAGGAGGGACATTGAGAG-3' | 504bp |
| hSHP-1-T7EN1 For2 | 5'-CTCTCTGCCTGCCCAGACTAG-3' | 579bp |
| hSHP-1-T7EN1 Rev2 | 5'-AGTTCTGGATCCGAATAT-3' | 579bp |

**Table S3. The oligonucleotide sequence for detecting potential off-target sites of SHP-1 sgRNA**

| **Primer name** | **Sequences** | **PCR products** |
| --- | --- | --- |
| hSHP-1-OFF1-For | 5'-CATGCTGTCAGTGCAGGCTG-3' | 470bp |
| hSHP-1-OFF1-Rev | 5'-GGCCCTCAGTGGGGAGCCCT-3' |  |
| hSHP-1-OFF2-For | 5'-TGGATCTGTCAGTCCTGGTT-3' | 489bp |
| hSHP-1-OFF2-Rev | 5'-ATGGGAACTCAAGCCTTGAC-3' |  |
| hSHP-1-OFF3-For | 5'-ACCACCGCCACCACTAAGCT-3' | 470bp |
| hSHP-1-OFF3-Rev | 5'-CAGCTGCTGGGAAGTCTCCG-3' |  |
| hSHP-1-OFF4-For | 5'-CTGGTATAAGATGATAACTC-3' | 454bp |
| hSHP-1-OFF4-Rev | 5'-AGAATTCTGTACCACCTATG-3' |  |

**Reference**

1. Yin AH, Miraglia S, Zanjani ED, Almeida-Porada G, Ogawa M, Leary AG, et al. AC133, a novel marker for human hematopoietic stem and progenitor cells. Blood. 1997,90(12):5002-12.
2. Zhu X, Prasad S, Gaedicke S, Hettich M, Firat E, Niedermann G. Patient-derived glioblastoma stem cells are killed by CD133-specific CAR T cells but induce the T cell aging marker CD57. Oncotarget. 2015,6(1):171-84.
3. Hu B, Zou Y, Zhang L, Tang J, Niedermann G, Firat E, et al. Nucleofection with Plasmid DNA for CRISPR/Cas9-Mediated Inactivation of Programmed Cell Death Protein 1 in CD133-Specific CAR T Cells. Hum Gene Ther. 2019,30(4):446-58.
